# Shuttle and stabilize: H1.2-FUS complex in amyotrophic lateral sclerosis pathogenesis

**DOI:** 10.4103/NRR.NRR-D-25-00422

**Published:** 2025-08-13

**Authors:** Dunja Petrovic, Gülce Perçin, David Vilchez

**Affiliations:** Institute for Integrated Stress Response Signaling, Faculty of Medicine, University Hospital Cologne, Cologne, Germany; Cologne Excellence Cluster for Cellular Stress Responses in Aging-Associated Diseases (CECAD), University of Cologne, Cologne, Germany; Institute for Genetics, University of Cologne, Cologne, Germany; Center for Molecular Medicine Cologne (CMMC), University of Cologne, Cologne, Germany

Amyotrophic lateral sclerosis (ALS) is a fatal, late-onset neurodegenerative disorder characterized by the progressive degeneration of motor neurons in the motor cortex, brainstem, and spinal cord (Feldman et al., 2022). ALS affects approximately 1.68 per 100,000 individuals worldwide, although its incidence varies across different populations (Feldman et al., 2022). The initial clinical manifestations typically include muscle twitching and weakness, which progressively lead to impairments in speech, swallowing, and respiration. Additionally, approximately half of ALS patients experience cognitive decline or behavioral changes, underscoring the broad spectrum of disease symptoms. The intricate interplay between genetic predisposition, environmental factors, and aging contributes to ALS pathogenesis, posing significant challenges for the development of effective therapies. Thus, a deeper understanding of the molecular and cellular mechanisms underlying ALS is essential for identifying targeted therapeutic strategies.

The etiology of sporadic ALS, which accounts for over 85% of cases, remains largely unknown, suggesting the involvement of multiple genetic variants in combination with additional risk factors (Feldman et al., 2022). The remaining cases follow a Mendelian pattern of inheritance. These familial ALS cases are associated with mutations in one of over 30 different genes, including several that encode RNA-binding proteins (Feldman et al., 2022). Among these, mutations in the fused in sarcoma (*FUS*) gene are among the most common causes of familial ALS (Feldman et al., 2022). FUS is predominantly a nuclear protein involved in DNA repair and RNA processing. However, non-synonymous mutations in its C-terminal nuclear localization signal, such as the aggressive P525L mutation, disrupt FUS nuclear transport, leading to its sequestration and aggregation in the cytoplasm (Vance et al., 2013; Feldman et al., 2022).

Notably, mutant FUS accumulates in stress granules (SGs), which are dynamic cytoplasmic RNA-protein assemblies that form in response to cellular stress when translation is stalled, thereby protecting RNA molecules from degradation (Vance et al., 2013; Alirzayeva et al., 2024). While SG assembly initially serves as a protective mechanism, their prolonged persistence can contribute to protein aggregation and cell death, which are hallmark features of many neurodegenerative diseases, including ALS (Wolozin and Ivanov, 2019; Gutierrez-Garcia et al., 2023). These findings suggest that FUS-ALS pathology is driven not only by a loss of nuclear FUS function but also by a toxic gain-of-function in the cytoplasm. Indeed, the incorporation of mutant FUS disrupts the dynamic properties of SGs, altering their assembly and clearance (Wolozin and Ivanov, 2019; Alirzayeva et al., 2024).

In a recent study, we used motor neurons derived from induced pluripotent stem cells (iPSCs) and *Caenorhabditis elegans* models to uncover molecular mechanisms underlying FUS^P525L^-driven pathology (Alirzayeva et al., 2024). Our findings provide deeper insight into the altered dynamics of the FUS^P525L^ interactome and its post-translational modifications (PTMs), while also identifying a previously unknown role of histone H1.2 in ALS pathogenesis. iPSC-derived motor neurons expressing endogenous levels of FUS^P525L^ provided a more physiologically relevant model for studying ALS-related changes in its interactome to previous approaches that relied on ectopic overexpression of mutant FUS. Moreover, the results were validated in motor neurons derived from patient iPSCs carrying other ALS-causing *FUS* mutations (R514S and R521C), reinforcing the broader implications of these pathogenic mechanisms.

The FUS^P525L^ interactome differs significantly from that of wild-type FUS, resulting in both loss and gain of protein interactors. Remarkably, mutant FUS exhibits increased interaction with histone H1.2, the only histone variant known to shuttle between the nucleus and cytoplasm (Lai et al., 2021). This gain of interaction influences FUS^P525L^ intracellular localization and aggregation, SG dynamics, and motoneuronal survival (Alirzayeva et al., 2024). The elevated interaction between H1.2 and FUS^P525L^ was observed exclusively in motor neurons, but not in their parental iPSCs, suggesting a reduced cellular capacity to maintain proteostasis following differentiation. Knockdown of H1.2 was sufficient to alleviate disease-related phenotypes in motor neurons, leading to predominantly nuclear localization of mutant FUS^P525L^, reduced cytoplasmic FUS aggregates, and restored SG dynamics. Conversely, H1.2 overexpression exacerbated ALS-related phenotypes (Alirzayeva et al., 2024).

PTMs, such as phosphorylation, ubiquitination, and poly(ADP-ribosyl)ation (PARylation), regulate protein-protein interactions and SG dynamics. For instance, ubiquitination and ubiquitin-like modifications modulate SG clearance via autophagy (Wolozin and Ivanov, 2019). PARylation, catalyzed by ADP-ribosyl transferase enzymes (PARPs), involves the transfer of poly(ADP-ribose) (PAR) to target proteins, playing roles in chromatin remodeling, DNA repair, and cell survival. The dysregulation of PARP enzymes, accompanied by elevated nuclear PARylation levels has been associated with ALS, suggesting a potential role in disease progression (McGurk et al., 2018). Furthermore, transient interactions between PAR and FUS — through an unknown mechanism — induce FUS condensation, leading to protein accumulation that mimics the ALS phenotype (Rhine et al., 2022).

Through a combination of approaches, we demonstrated that mutant FUS^P525L^-H1.2 interaction is regulated by PARylation and promotes FUS^P525L^ accumulation in the cytoplasm (Alirzayeva et al., 2024). Importantly, pharmacological inhibition of PARylation via olaparib weakened the FUS^P525L^-H1.2 interaction and reduced motoneuronal degeneration, while PDD0017273, an inhibitor of poly(ADP-ribose) glycohydrolase, exacerbated FUS^P525L^ aggregation and apoptosis. These findings highlight a critical role of PARylation in FUS^P525L^ accumulation mediated by H1.2.

The relevance of the FUS^P525L^-H1.2 interaction in ALS pathology was further validated in vivo using *Caenorhabditis elegans* models. Expression of human FUS^P525L^ in the *Caenorhabditis elegans* nervous system leads to age-dependent FUS aggregation, reduced motility, and shortened lifespan, replicating disease-related phenotypes. Notably, these pathological phenotypes were rescued by downregulation of H1.2 and PARP1 orthologs. Conversely, neuron-specific overexpression of H1.2 worsened ALS-related phenotypes in ALS worm models (Alirzayeva et al., 2024).

**Figure 1:**
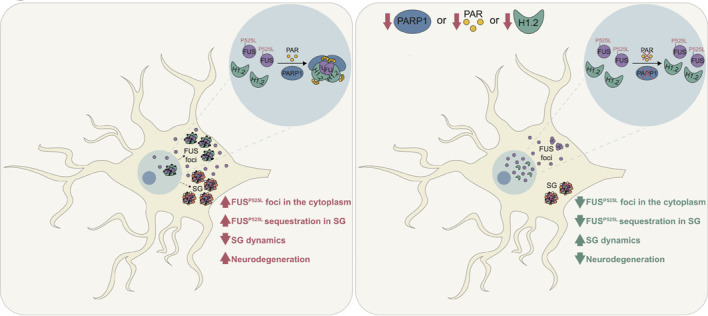
Proposed model of H1.2 and PARylation-driven FUS^P525L^ toxicity in motor neurons. The increased interaction between mutant FUS^P525L^ and H1.2, stabilized by PARylation, promotes the cytoplasmic accumulation of FUS, its enhanced incorporation into SGs, and altered SG dynamics, ultimately leading to neuronal cell death. Targeting PARylation through PARP1 inhibition or reducing H1.2 levels mitigates disease-related phenotypes by decreasing FUS^P525L^ toxicity. This schematic was generated based on data from Alirzayeva et al. (2024). Neuronal morphology elements were adapted from Servier Medical Art (https://smart.servier.com/citation-sharing/) and all other components were created in Adobe Illustrator. FUS: Fused in sarcoma; H1.2: histone H1.2; PAR: poly(ADP-ribose); PARP1: poly(ADP-ribose) polymerase 1; SG: stress granule.

Together, our study identifies a role for histone H1.2 in FUS-ALS pathology, demonstrating its involvement in cytoplasmic FUS mislocalization and aggregation through a PARylation-dependent mechanism (**Figure 1**). These findings provide new insights into ALS pathogenesis and suggest potential therapeutic strategies targeting histone dynamics and PARylation to mitigate disease progression, while also raising new questions for future investigation.

For instance, further research is needed to elucidate the factors that modulate FUS-H1.2 interaction in the context of ALS. FUS oligomerization is primarily driven by its N-terminal low-complexity domains and is enhanced by nucleating molecules such as RNA or PAR (Rhine et al., 2022). Similarly, histone H1.2 possesses disordered N-terminal and C-terminal domains, which we found to be required for FUS^P525L^-H1.2 interaction (Alirzayeva et al., 2024). However, it remains unclear which FUS domains mediate interactions with H1.2 and how ALS-related mutations influence FUS conformation and its affinity for H1.2.

Both FUS and H1.2 undergo extensive PTMs. PARylation, a key mechanism in PARP1-dependent FUS condensation at DNA damage sites, is typically reversible and does not normally lead to insoluble nuclear FUS aggregates (Singatulina et al., 2019). While *in vitro* studies suggest that PAR is a more potent nucleator of FUS compared to RNA, the interplay between low intracellular PARylation levels, its rapid and reversible nature, and competition with RNA molecules may be involved in fine regulation of FUS interactome and cellular functions (Rhine et al., 2022). Based on our findings, we speculate that elevated total PARylation levels, along with the increased affinity of mutant FUS for PAR, may drive its aberrant aggregation and promote FUS binding to H1.2 through the acquisition of additional interactions or the stabilization of transient interactions typical of wild-type FUS.

Further *in vitro* studies using recombinant proteins could help clarify the molecular mechanisms and specific regions involved in FUS-H1.2 interactions. For instance, experiments incorporating FUS mutations that affect cellular localization or putative interacting domains, combined with different nucleators such as PAR and RNA, may provide deeper insights into the dynamics of FUS-H1.2 interactions under various cellular conditions. Moreover, beyond PARylation, other PTMs (e.g., H1.2 phosphorylation and the previously characterized arginine methylation of FUS) could modulate FUS-H1.2 interactions, further contributing to the complex pathological mechanisms underlying ALS.

Notably, most pathological features observed in our study were either more pronounced (e.g., alterations in SG dynamics) or exclusively detectable in motor neurons (e.g., the gain of interaction of mutant FUS with H1.2), but not in their parental iPSCs (Alirzayeva et al., 2024). This suggests a potential explanation for the higher sensitivity to FUS-induced toxicity in motor neurons. While motor neurons remain the primary focus of ALS research, a study indicates that microglial dysfunction can also contribute to disease progression and neuronal pathology (Clarke and Patani, 2020). Therefore, it would be valuable to investigate whether PARylation-mediated FUS-H1.2 complexes are specific to neurons or if they represent broader ALS-associated hallmarks across different cell types.

Beyond familial cases linked to *FUS* mutations, cytoplasmic inclusions of wild-type FUS are frequently observed in sporadic ALS cases. This suggests that while FUS mislocalization and aggregation may not always be the primary trigger of disease onset, they likely play a role in disease progression. Based on our data, it would be of interest to investigate whether FUS-positive cytoplasmic foci in sporadic ALS result from PTM-driven alterations in the FUS interactome. To address this, proteomic analysis of the interactome of wild-type FUS and its PTMs in iPSC-derived motor neurons from sporadic ALS patients could provide valuable insights. If wild-type FUS has aberrant PARylation and enhanced interaction with histone H1.2 similar to mutant FUS, the next step would be to determine whether these alterations contribute to sporadic ALS pathology, including FUS aggregation and neurodegeneration.

In addition to ALS, we speculate that the interaction between FUS and histone H1.2 may also play a role in frontotemporal dementia (FTD). FTD largely overlaps with ALS at the genetic level and shares key pathological and molecular features, including alterations in SG dynamics (Feldman et al., 2022). Approximately 50% of ALS patients develop cognitive and behavioral symptoms associated with FTD, while 30% of FTD patients present with motor symptoms characteristic of ALS. Thus, FTD and ALS are considered to represent two ends of a spectrum disorder (Feldman et al., 2022). Given that FUS mutations and dysregulation are implicated in both disorders, it is plausible that increased FUS PARylation and interaction with H1.2 may contribute to SG dysregulation and neurodegeneration in FTD as well. As with sporadic ALS, iPSC-derived neurons from FTD patients could provide a valuable model to investigate this hypothesis.

*This work was supported by the Else Kröner-Fresenius-Stiftung (2021-EKSE.95), and the Deutsche Forschungsgemeinschaft (CRC1678 and Germany’s Excellence Strategy-CECAD, EXC 2030-390661388) (to DV).*
